# Author Reply to Letter to the Editor: “The elusive predictors of anterior cruciate ligament failure in the young: Null findings in registry data”

**DOI:** 10.1002/ksa.70279

**Published:** 2026-01-13

**Authors:** Riccardo Cristiani, Karl Eriksson

**Affiliations:** ^1^ Department of Molecular Medicine and Surgery, Section of Sports Medicine Karolinska Institutet Stockholm Sweden; ^2^ Stockholm Sports Trauma Research Center (SSTRC) FIFA Medical Centre of Excellence Stockholm Sweden; ^3^ Department of Orthopaedics Stockholm South Hospital Stockholm Sweden; ^4^ Karolinska Institutet Stockholm Sweden

To the Editor,

We appreciate the authors' interest in our study [1]. However, after carefully reviewing their letter [3], we believe several points require clarification. We would like to kindly invite the authors to revisit our article with close attention, as their comments appear to be based on fundamental misunderstandings of the content.

First, the study does not present any concern regarding Type II error. The actual revision event rate was 9.0% (*n* = 170) within a cohort of 1888 patients, and not 4.2% (*n* = 100) in 2374 patients as stated by the authors. The statistical methods used—univariable analyses and multivariable logistic regression—are entirely appropriate for the dataset. While no strict rule exists, a commonly accepted guideline is to include no more than *n*/10 variables in multivariable regression, where *n* is the number of events [2]. Our multivariable logistic regression model included five independent variables, which is well within the ‘permissible’ range given the substantial number of events in our study.

Second, there was no aggregation of disparate surgical techniques (transphyseal, all‐epiphyseal, or hybrid), as suggested by the authors. As clearly stated in the first sentence of the Surgical Technique and Rehabilitation section, all patients underwent transphyseal anterior cruciate ligament reconstruction (ACLR). Furthermore, contrary to what is reported in the letter, detailed information on graft fixation methods is explicitly provided. Femoral fixation was performed using either an EndoButton (Smith & Nephew) or a TightRope (Arthrex) device, while tibial fixation was achieved using Ethibond no. 2 sutures (Ethicon) tied over an AO bicortical screw with a washer. We fully acknowledge the well‐established relationship between tunnel positioning and graft failure or revision surgery. However, radiographic assessment of tunnel placement is not feasible in a cohort of more than 1800 patients. Importantly, all procedures were performed at a single, high‐volume, highly specialised arthroscopic center by experienced knee surgeons, which minimises the potential for confounding related to tunnel positioning.

Third, the definition of the outcome—revision ACLR—should not be viewed as a limitation of the study. We acknowledge that revision ACLR is not synonymous with graft failure and that some patients who experienced graft failure (a concept for which multiple definitions exist) might not have been captured. However, it is equally important to note that revision ACLR represents a clinically meaningful and objectively verifiable endpoint, reflecting a degree of knee dysfunction significant enough to warrant surgical intervention. Moreover, in children and adolescents, who typically have high functional demands, it is reasonable to assume that the vast majority of graft failures would lead to revision surgery and therefore be captured by our definition. Graft failure and revision ACLR are not hierarchical outcomes; they simply represent different endpoints, each with its own strengths and limitations. No additional outcomes, such as patient‐reported measures, were included because the explicit aim of the study was to evaluate revision ACLR, and the analyses were designed accordingly.

Fourth, the study does not omit functional data related to return‐to‐sport readiness. On the contrary, isokinetic knee extension and flexion strength, as well as single leg hop test performance, were explicitly included as independent variables in our analyses. We kindly encourage the authors of the letter to review the Materials and Methods and the Results sections—particularly Tables 1 and 2—where these data are clearly reported. Therefore, the assertion that the ‘study's framework omits the modern paradigm of dynamic risk, which is defined by return‐to‐sport readiness… and that… the absence of this functional data creates a critical gap in the analytical model, leaving the most probable drivers of failure unmeasured and unaccounted for’ is not factually correct.

In conclusion, we respectfully—but firmly—disagree with the authors' statements that our null findings ‘may stem from limited power, confounding from combining heterogeneous techniques, and omission of key functional risk factors like neuromuscular readiness’ as well as their claim that our study ‘underscores the limitations of a registry‐based analysis that lacks granular surgical data, functional outcomes, and is potentially underpowered for its primary aim’. As detailed above, and as is clearly documented in our manuscript [1], these assertions are not supported by the study design, the data included, or the analyses performed.

We thank the authors once again for their thoughtful input and cordially invite them to revisit our article in light of the clarifications outlined in this response.
